# Ambulatory monitoring demonstrates an acute association between cookstove-related carbon monoxide and blood pressure in a Ghanaian cohort

**DOI:** 10.1186/s12940-017-0282-9

**Published:** 2017-07-21

**Authors:** Ashlinn K. Quinn, Kenneth Ayuurebobi Ae-Ngibise, Patrick L. Kinney, Seyram Kaali, Blair J. Wylie, Ellen Boamah, Daichi Shimbo, Oscar Agyei, Steven N. Chillrud, Mohammed Mujtaba, Joseph E. Schwartz, Marwah Abdalla, Seth Owusu-Agyei, Darby W. Jack, Kwaku Poku Asante

**Affiliations:** 1 Department of Environmental Health Sciences, Mailman School of Public Health, Columbia University, 722 West 168th St, 11th floor, New York, 10032 NY USA; 2 Kintampo Health Research Centre, Ghana Health Service, Brong Ahafo Region, Kintampo, Ghana; 3 Department of Environmental Health, Boston University School of Public Health, Boston, MA USA; 4 Division of Maternal-Fetal Medicine, Vincent Department of Obstetrics and Gynecology, Massachusetts General Hospital and Harvard Medical School, Boston, MA USA; 5 Department of Medicine, Columbia University Medical Center, New York, NY USA; 6 Lamont-Doherty Earth Observatory of Columbia University, Palisades, NY USA; 7 Institute for Applied Behavioral Medicine Research, Stony Brook University, Stony Brook, NY USA; 8 Center for Behavioral Cardiovascular Health, Columbia University, New York, NY USA

**Keywords:** Household air pollution, Carbon monoxide, Blood pressure, Ambulatory blood pressure monitoring, Cookstoves, Biomass

## Abstract

**Background:**

Repeated exposure to household air pollution may intermittently raise blood pressure (BP) and affect cardiovascular outcomes. We investigated whether hourly carbon monoxide (CO) exposures were associated with acute increases in ambulatory blood pressure (ABP); and secondarily, if switching to an improved cookstove was associated with BP changes. We also evaluated the feasibility of using 24-h ambulatory blood pressure monitoring (ABPM) in a cohort of pregnant women in Ghana.

**Methods:**

Participants were 44 women enrolled in the Ghana Randomized Air Pollution and Health Study (GRAPHS). For 27 of the women, BP was measured using 24-h ABPM; home blood pressure monitoring (HBPM) was used to measure BP in the remaining 17 women. Personal CO exposure monitoring was conducted alongside the BP monitoring.

**Results:**

ABPM revealed that peak CO exposure (defined as ≥4.1 ppm) in the 2 hours prior to BP measurement was associated with elevations in hourly systolic BP (4.3 mmHg [95% CI: 1.1, 7.4]) and diastolic BP (4.5 mmHg [95% CI: 1.9, 7.2]), as compared to BP following lower CO exposures. Women receiving improved cookstoves had lower post-intervention SBP (within-subject change in SBP of −2.1 mmHg [95% CI: -6.6, 2.4] as compared to control), though this result did not reach statistical significance. 98.1% of expected 24-h ABPM sessions were successfully completed, with 92.5% of them valid according to internationally defined criteria.

**Conclusions:**

We demonstrate an association between acute exposure to carbon monoxide and transient increases in BP in a West African setting. ABPM shows promise as an outcome measure for assessing cardiovascular health benefits of cookstove interventions.

**Trial registration:**

The GRAPHS trial was registered with clinicaltrials.gov on 13 April 2011 with the identifier NCT01335490.

**Electronic supplementary material:**

The online version of this article (doi:10.1186/s12940-017-0282-9) contains supplementary material, which is available to authorized users.

## Background

Household Air Pollution (HAP) from cooking using biomass fuels has been estimated to be the fourth leading risk factor for death and disability globally [1] and the leading risk factor in Ghana [2]. A significant proportion of this risk is attributable to adult cardiovascular disease (CVD). Despite the fact that the long latency of CVD provides a challenge to studies of the association between exposure to HAP and CVD clinical outcomes, several observational studies have reported positive associations between the use of polluting fuels (biomass/kerosene/diesel) and cardiovascular diagnoses and/or CVD mortality [3–5]. Increased blood pressure (BP) is one mechanism thought to potentially underlie the relationship between household air pollution exposure and cardiovascular disease, due to existing evidence of an association between ambient particulate matter (PM) exposure and increased BP [6], and a large body of literature linking increased BP to cardiovascular events and mortality [7]. The specific mechanism by which PM exposure may lead to increased BP remains unknown, but has been hypothesized to involve vasoconstriction, endothelial dysfunction, and/or autonomic nervous system imbalance [6].

Studies of the association between HAP and BP/hypertension are proliferating: there are currently published manuscripts concerning this association from cohorts in Guatemala [8], India [9–11], Nicaragua [12], China [3, 13], and Peru [14, 15]. The studies differ in the methods used to categorize HAP exposure. Many studies employed relatively crude indicators of exposure to HAP, for example “ever” use of biomass fuel over the life course [3, 14], or current use of solid fuel [11]. Fewer studies actually measured pollutants in locations such as kitchens and living areas or using personal exposure monitoring [9, 10, 12, 16, 17].

Carbon monoxide (CO) is a component of household air pollution [18] that is more easily and inexpensively measured than PM_2.5_ and has been commonly measured in studies of the health effects of HAP, although reported correlations with PM are variable across settings. Some of the largest correlations have been reported in settings where both pollutants are largely derived from biomass burning, e.g. households in rural Peru, The Gambia, and Guatemala [19–21], while lower correlations have been reported in other rural settings, possibly due to exposure to multiple sources of pollution [22, 23]. CO itself may acutely affect BP: a study among Brazilian traffic controllers found increases in BP associated with same-day elevations in ambient CO [24]. The Ghana Randomized Air Pollution and Health Study (GRAPHS) study, a cookstove intervention trial among 1414 pregnant women, measured HAP using personal CO monitoring supplemented with occasional personal PM_2.5_ monitoring [25]. We have previously reported an association between mean 72-h CO exposure and BP in the GRAPHS cohort at trial enrollment [17]; this analysis was cross-sectional and did not investigate acute effects of CO on BP.

As a clinical biomarker of CVD risk, BP has the advantage of being non-invasive and inexpensive. Traditional “clinic” measurements of BP, however, are imperfect estimates of BP, as BP levels vary throughout the day and may be influenced by the clinic environment in either an upward or downward direction [26]. Alternative methods of measuring BP that overcome these shortcomings are ambulatory blood pressure monitoring (ABPM) and home blood pressure monitoring (HBPM). Both ABPM and HBPM have been shown in numerous studies to correlate with CVD and mortality outcomes, independently of clinic-measured BP [27]. In ABPM, a portable monitor and blood pressure cuff are worn for an extended period (typically 24 h), with BP measurements taken at approximately 15 to 30 min intervals throughout the day and the night. In HBPM, a semi-automatic digital BP monitoring device is used to measure BP repeatedly in the home setting over the course of several consecutive days. ABPM currently is considered the gold standard for accurately assessing blood pressure since it provides a comprehensive assessment of an individual’s complete BP profile, including the diurnal variation that may be important to cardiovascular risk [28]. Many American and European clinical guidelines recommend 24-h ABPM for the diagnosis and treatment of hypertension [27]. Despite its clear clinical potential, ABPM is relatively underutilized in low and middle income countries [29]. One study has reported using ABPM to measure BP in association with HAP exposure in India; this study investigated associations during cooking sessions only and was inconclusive in its findings [10]. We know of no studies that have reported on the feasibility of ABPM in a rural African setting.

Here, we use ABPM among a subset of pregnant women enrolled in GRAPHS to investigate two hypotheses: first, that acute exposure to CO would trigger acute increases in BP; and second, that cookstove interventions to reduce exposure to HAP would lead to lower post-intervention BP as compared to control.

## Methods

### Study Participants

This study was carried out in a subset of women (*n* = 44) enrolled in the GRAPHS trial, which has been described previously [25]. In short, GRAPHS is a community-level randomized controlled cookstove intervention trial among pregnant women living in rural communities of the Kintampo North and Kintampo South districts, located in a predominantly rural area in the middle belt of Ghana. GRAPHS women are non-smokers, the primary cooks for their households, and at less than 28 weeks of gestation at the time of enrollment as established by ultrasound [30]. Ethical approvals for this study were obtained from the Institutional Review Board of Columbia University Medical Center and the Kintampo Health Research Centre Institutional Ethics Committee. Informed consent was obtained from all human subjects before the research began.

Women were recruited into this BP study between July and November 2014, at the time of their enrollment into the parent GRAPHS trial. The selected women were a convenience sample chosen such that half the women were living in intervention communities and half in control communities. In this region, the traditional cookstove is a “three-stone” open fire fueled by firewood. Women in control communities continued cooking with traditional fires throughout the trial, which spanned the remainder of the woman’s pregnancy and through the infant’s first year of life. Women in intervention communities received improved cookstoves one to 3 weeks following enrollment into GRAPHS, with the intervention cookstoves being either: a) improved-combustion biomass-burning BioLite HomeStoves (BioLite Inc., Brooklyn NY); or, b) two-burner liquefied petroleum gas (LPG) stoves and associated cooking fuel. Women were encouraged to complete all cooking activities using the new stoves for the duration of the trial, and research workers tracked their stove use weekly.

### BP Measurement Protocols

Each woman’s BP was measured during two distinct sessions. The first measurement occurred within a week of enrollment into GRAPHS, prior to the delivery of improved cookstoves to the women in intervention communities. All women were thus still cooking on traditional fires at the time of this “baseline” BP measurement session. The second BP measurement session occurred approximately 3–4 weeks later, after the initiation of cooking with improved cookstoves in the intervention groups.

We designed two BP measurement protocols for this study. The first employed 24-h ABPM to measure BP. SpaceLabs 90,207 ABP monitors (SpaceLabs Medical, Inc.; Redmond, WA) were deployed to the study participants for 24-h periods, as is commonly done in clinical practice [26]. These monitors have been validated according to the standards of the Association for the Advancement of Medical Instrumentation [31, 32]. Trained research staff deployed the monitors, which were set to record BP every 20 min during the daytime and every 30 min during the nighttime. The research workers returned approximately 24 h later to retrieve the monitors from the women and to download the data. Women self-reported their sleep and wake times during the monitoring period.

We developed a second parallel BP protocol using home blood pressure monitoring to compare with the ABPM arm as a secondary aim of the study was to assess the feasibility of ABPM in this setting. European Society for Hypertension (ESH) guidelines for HBPM recommend that BP be measured morning and evening for a minimum of 3–4 consecutive days with at least two BP measurements taken per occasion [26]. Here, we employed Omron BP791IT automatic digital blood pressure monitors (Omron Healthcare, Inc.; Bannockburn, IL) to measure BP each morning and evening over the course of five consecutive days. These monitors have been validated against mercury sphygmomanometers for the general adult population according to the European Society of Hypertension International Protocol [33]. Trained resident fieldworkers operated the monitors, while the women were seated and resting with the BP cuff attached to the left arm. At each morning and evening visit, the fieldworkers had the participant rest in a seated position for 5 min, then operated the BP monitors using a setting that automatically measured BP three times, spaced apart by 1 min of rest. The automatic setting generated the average systolic and diastolic BP (SBP; DBP) over the three measurements (individual measurements were not retained).

**Fig. 1:**
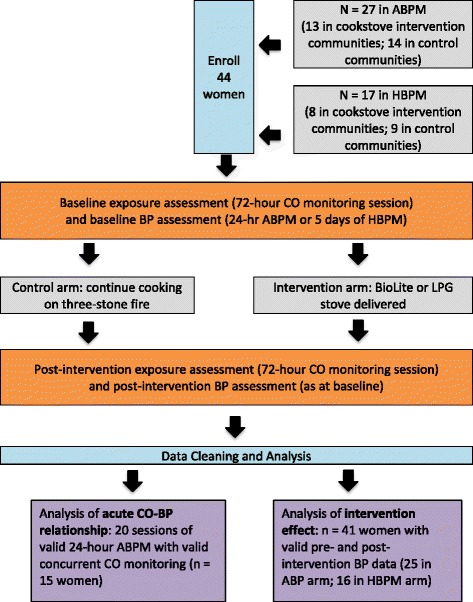
Flow chart of study activities

Participants were enrolled into either the ABPM or the HBPM arm of the study using a convenience approach, and the same protocol (either ABPM or HBPM) was used for the first (baseline) and second (post-intervention) BP measurement sessions for each woman. Figure 1 shows a visualization of the workflow of the two BP measurement protocols.

### BP validity

Validity for 24-h ABPM was calculated using the criteria of the International Database of Ambulatory Blood Pressure in relation to Cardiovascular Outcome (IDACO) project [34], which specifies a minimum of ten successful BP measurements during waking hours and a minimum of five successful BP measurements during the sleep period. No additional validation of HBPM was conducted beyond the automatic procedures inherent to the devices, which report errors if BP measurement is disturbed by movement or other factors.

### BP outcome definitions

In the HBPM arm, mean SBP and DBP were calculated by taking the average of all recorded SBP/DBP measurements over the course of each 5 day-session. Hypertension in the HBPM arm was defined per ESH recommendations [26]. In the ABPM arm, 24-h BP was calculated from the valid sessions using the mean of all successful BP recordings during the monitored period. Additionally, awake and asleep SBP and DBP were calculated in this arm, using the average of the measurements taken during the women’s self-reported sleeping and waking periods. Definitions for specific BP phenotypes follow the recommendations of the ESH [35] and are provided in Table 1.

**Table 1:** BP definitions, per European Society of Hypertension

HBPM	Hypertension	Mean SBP ≥ 135 mmHg or mean DBP ≥ 85
ABPM	Hypertension	Mean 24 h SBP ≥ 130 mmHg or mean 24 h DBP ≥ 80 mmHg
	Awake ambulatory hypertension	Mean awake SBP ≥ 135 mmHg or mean awake DBP ≥ 85 mmHg
	Nocturnal hypertension	Mean asleep SBP ≥ 120 mmHg or mean asleep DBP ≥ 70 mmHg
	Non-dipping pattern	Mean asleep SBP ≤ 10% lower than mean awake SBP

### Exposure monitoring

Personal CO measurements were obtained using Lascar EL-USB-CO monitoring devices (Lascar Electronics, Erie, PA), deployed for a 72-h period following the GRAPHS trial protocol [25]. These monitors record real-time CO exposure in parts per million (ppm), with measurements taken every 10 seconds. The timing of the BP measurement sessions was designed to coincide with the three-day CO monitoring sessions. While personal PM exposure was concurrently collected in some sessions per the GRAPHS protocol, we had too few valid PM monitoring sessions in our sample to enable use of the PM data in our analyses, and restricted our analyses to CO.

We generated hourly averages of CO exposure and used these values to define exposure in our subsequent models: first employing them as continuous variables of exposure; and then creating binary variables to indicate exposure above thresholds. The thresholds of “peak” CO exposure were defined using percentiles of the exposure distribution.

### Data analysis

#### Acute CO-BP effect

Analysis of the acute CO-BP effect employed data from the women whose BP was measured using 24-h ABPM. We averaged BP measurements by hour, and analyzed hourly SBP and DBP as outcomes in separate multilevel linear regression models, with the 24-h ABP monitoring session as the grouping variable. The models were adjusted for the awake period (based on each individual’s self-reported waking hours), when BP is known to be higher than during sleep, and for the two-hour peri-waking period of the morning, when BP rises rapidly [27]. Other potential individual and time-varying covariates, such as hour of day, day of week, age, gestational age, socioeconomic status, and BMI, were tested for inclusion in the models but did not affect our main estimates of interest and thus were not retained in the final models.

**Fig. 2:**
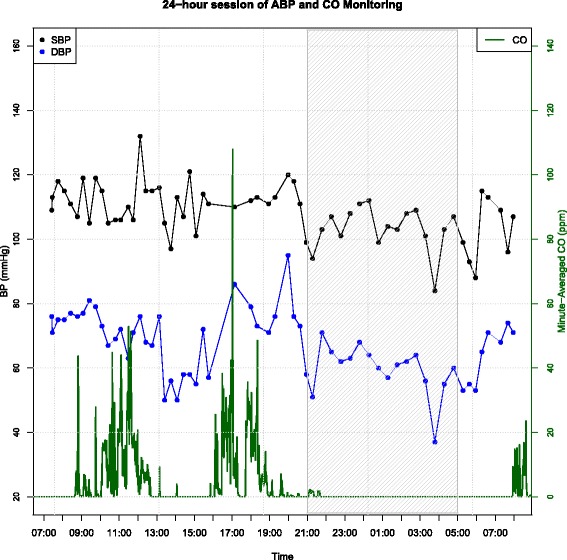
Example data from 24-h ABPM and concurrent CO exposure monitoring session. *Black dots* and lines: SBP (mmHg). *Blue dots* and *lines*, DBP (mmHg). *Green lines*: minute-averaged CO (ppm). Grey hatching indicates the sleep period

We evaluated the association between CO exposure and BP across our sample in two ways: first, using multilevel regression models to evaluate the impact of same-hour and lagged mean hourly CO on BP across our dataset; and second, evaluating the impact of CO exposure above high thresholds on subsequent BP measurements. We chose to evaluate thresholds because CO exposure in the cooking setting is dominated by long periods of low exposure with episodic spikes ([36, 37], see Fig. 2), and we hypothesized that BP would respond to higher CO exposure.

To analyze the threshold effect we calculated the percentiles of CO exposure across our monitored population, and defined “peak” CO as exposure at or above the 90th percentile of the distribution of a two-hour CO moving average: this value was 4.1 ppm. The choice of a two-hour moving average was grounded in experimental and chamber studies that have demonstrated an association between acute PM exposure and increased BP within about 2 h following PM exposure [38–41], and by confirmation in our data that BP was increased at lags 0 and 1 following CO exposure, but that the association went away at lag 2: results of initial exploratory models using each hour of CO (lag 0, lag 1, and lag 2) separately and combined can be found in the Additional file 1: Table S1.

Peak CO was entered in the models as a fixed binary predictor indicating whether or not the two-hour moving average of CO prior to BP measurement was above 4.1 ppm. A potential limitation of this approach is that CO exposure for some exposure monitoring sessions never crossed the 90th-percentile threshold and these data were therefore excluded. To address this potential shortcoming, we conducted two sensitivity analyses using alternate definitions of CO. In the first sensitivity analysis, we continued to pool the measured CO values across our study population but chose different percentiles (75th, 80th, 85th, and 95th) for our definition of “peak” CO exposure.

As a second sensitivity analysis, we used each individual 24-h session of CO measurements to define “peak” CO, again using the 75th to 95th percentiles (rather than pooling the exposure measurements across all the CO monitoring sessions to determine the percentiles). Every individual thus had some hours of the day that met the criteria for “peak” CO exposure. While it is more difficult to generate generalizable thresholds of CO using these individually-determined definitions, this analysis speaks to whether a woman’s BP levels were higher than usual following her own highest daily exposure of CO.

The final models for the acute CO-BP effect were two-level random intercept linear regression models with the form:

Level 1: (hourly BP measurements)

$$ {\mathrm{BP}}_{\mathrm{i}\mathrm{j}}{=\upbeta}_{0\mathrm{j}}{+\upbeta}_1{\mathrm{CO}}_{\mathrm{i}}{+\upbeta}_2{\mathrm{T}}_{\mathrm{i}}{+\upvarepsilon}_{\mathrm{i}\mathrm{j}} $$

Level 2: (24-h BP session)


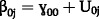


Where:

- The outcome variable, BP, represents hourly-averaged SBP or DBP for hour *i* within ABPM session *j*.
- In models using CO as a continuous variable, the primary exposure, CO, indicates hourly CO exposure at hour *i* or lagged by 1–8 h prior to hour *i*. In analyses of the threshold effect, the primary exposure, CO, is a binary variable indicating exposure at or above the “peak” CO level in the 2 h previous to hour *i*.
- The vector T represents a matrix of covariates that vary at the same level as the individual BP measurements (Level 1); that is, in time. In this case T contains indicator variables for the awake period and for the morning period.
- The intercept, β_0j_, contains an error term, U_0j_, allowing the intercept to vary across BP sessions (Level 2).
- ℇ_ij is_ the within-session variation not explained by the predictors CO and T.

An AR1 correlation structure was included in the models to account for hourly autocorrelation in BP values.

#### Intervention effect

To test our second hypothesis that a cookstove intervention would be associated with reduced BP, we combined data from the women monitored with both BP protocols (ABPM and HBPM). We designated a single intervention group for women receiving any type of improved cookstove (whether BioLite or LPG). We calculated the change in awake SBP and awake DBP between the first and second BP monitoring sessions for each woman, and compared these values between the control and intervention groups using linear regression models. We considered the following individual-level characteristics for inclusion in the models due to their known associations with BP: age, BMI, gestational age, and reported stress level. Of particular interest was gestational age, since BP is known to change across the time course of pregnancy [42]. The other variables, while potentially associated with an individual’s BP level, would not be expected to influence the within-person change in BP as measured across two sessions 3 weeks apart and were not included in the models. We also included the type of BP assessment (ABPM versus HBPM) in the model as a precision variable. The final model took the form:

$$ {\mathrm{BPCHANGE}}_{\mathrm{i}}{=\upbeta}_0{+\upbeta}_1{\mathrm{INT}}_{\mathrm{i}}{+\upbeta}_2{\mathrm{GESTWKS}}_{\mathrm{i}}{++\upbeta}_3{\mathrm{BPTYPE}}_{\mathrm{i}}+\upvarepsilon $$

Where the outcome, BPCHANGE, is calculated as post-intervention SBP (or DBP) minus baseline SBP (or DBP) for each individual *i*; INT is intervention status, GESTWKS is weeks of gestation at enrollment, and BPTYPE is an indicator for ABPM or HBPM monitoring.

All data analysis was conducted using R version 3.0.2 (R Foundation for Statistical Computing, Vienna, Austria).

## Results

Forty four pregnant women were successfully enrolled into the study (27 in the ABPM group; 17 in the HBPM group). None of the women had been previously diagnosed as hypertensive or diabetic. The women were on average 16 to 17 weeks gestation at the time of enrollment and were in their mid- to late twenties (Table 2).

**Table 2:** Baseline characteristics of 44 women enrolled in the BP study, compared to all women in the GRAPHS cohort

	ABPM group	HBPM group	All GRAPHS women
Number of participants enrolled	27	17	1414
Number of intervention participants	13	8	
Improved biomass (BioLite™) stove	5	0	
Liquefied Petroleum Gas (LPG) stove	8	8	
Age (years)^a^	23.9 [6.5]	29.4 [8.4]	27.5 [7.1]
BMI (kg/m^2^)^a^	21.8 [1.8]	22.8 [3.1]	23.4 [3.2]
Gestational age (weeks, at enrollment)^a^	16.6 [4.3]	16.9 [4.8]	16.2 [4.4]
Pre-intervention mean CO exposure (ppm)^a^	1.43 [0.75]	1.12 [0.79]	1.76 [1.1]
Pre-intervention SBP (mmHg)^a^	106.9 [6.4]	100.6 [9.4]	105.5 [10.2]
Pre-intervention DBP (mmHg)^a^	62.6 [4.8]	65.8 [6.2]	63.2 [7.9]

^a^Continuous variables reported as mean [SD]

### Acute effect of CO on BP

For the acute CO-BP analysis we included any 24-h ABPM sessions that had been conducted concurrently with personal CO monitoring (defined as CO monitoring that overlapped by 20 h or more with the ABP monitoring). Twenty ABPM sessions, among 19 women, met this criterion. An example is shown in Fig. 2.

Our initial modeling evaluated the association between 0- to 8-h-lagged CO exposure (as a continuous variable) and BP. We did not observe a significant relationship between CO and BP in these models, although the coefficients were in the expected direction for the one-hour-lagged CO variable (Additional file 1: Figure S1).

In our analyses of a potential threshold effect of high CO exposure on subsequent BP, we found that peak CO exposure was significantly associated with increases in both SBP and DBP (Table 3; full model output available in the Additional file 1: Table S2). On average, exposure above 4.1 ppm was associated with an increase in hourly-averaged SBP of 4.3 [1.1, 7.4] mmHg and an increase in hourly-averaged DBP of 4.5 [1.9, 7.2] mmHg as compared to BP following lower CO exposures, adjusted for awake and morning periods.

**Table 3:** Association of hourly peak CO exposure with hourly SBP and DBP

	SBP (mmHg)	DBP (mmHg)
Coefficient for Peak CO^a^ [95% CI]	4.3 [1.1, 7.4]	4.5 [1.9, 7.2]

^a^The coefficient is the estimated fixed-effect difference in BP associated with peak hourly CO exposure (defined as the 90th percentile of a two-hour moving average of CO, or 4.1 ppm) versus all other CO exposure, adjusted for self-reported awake hours and for the 2-h peri-waking “morning” period. Model is a multilevel linear regression model with a random intercept for each 24-h monitoring session. Estimates incorporate an autoregressive (AR1) correlation structure. *n* = 20 CO and BP monitoring sessions among 19 women

**Fig. 3:**
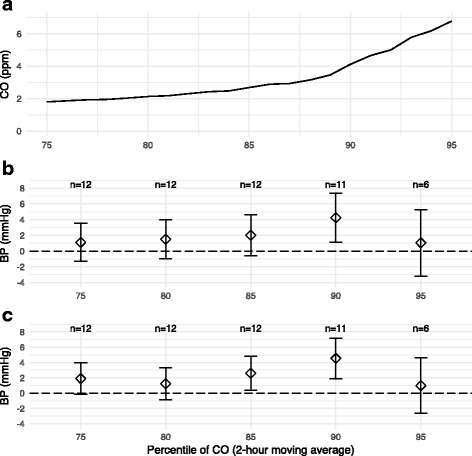
**a** 75th–95th percentiles of 2-h moving average of CO across 20 24-h personal monitoring sessions. **b** Beta coefficients for effect of CO exposure above 75th–95th percentile thresholds on hourly SBP, with thresholds determined using the distribution of CO exposure across all 20 monitoring sessions, N indicates the number of monitoring sessions included in the model for each percentile. **c** as **b**, but for DBP

Not all 20 CO monitoring sessions contained exposure values meeting our definition of “peak” CO exposure: two-hour moving average of CO exceeding the 90th percentile of the distribution (4.1 ppm). In all, 12 out of 20 (60%) of CO sessions contained values exceeding the 90th percentile. Because this definition excluded a large percentage of the data, we conducted several sensitivity analyses (see methods). First, by defining “peak” CO alternatively as the 75th, 80th, 85th, and 95th percentiles of the exposure distribution, we observed that SBP/DBP increased as the CO percentile increased (Fig. 3). The exception was peak CO defined using the 95th percentile definition, although this model was impacted by a lack of data: only seven sessions (35%) contained CO exposure levels exceeding the 95th percentile value (2-h moving average above 6.8 ppm).

**Fig. 4:**
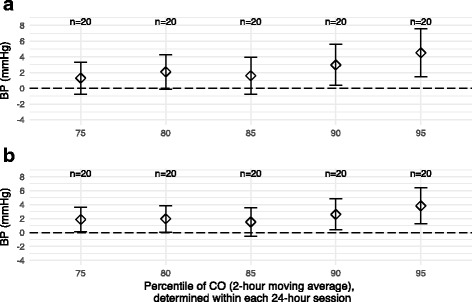
Beta coefficients for effect of CO exposure on above 75th–95th percentile thresholds on hourly BP, with thresholds determined within each monitoring session (*N* = 20 for all percentiles, indicating all data was included in each model). Multilevel regression models were adjusted for awake time and morning period, with 24-h ABPM monitoring session as the grouping variable. Error bars indicate the 95% CI for the beta coefficient. **a** SBP; **b** DBP

A second sensitivity analysis, using each individual 24-h session of CO measurements to define individually-specific “peak” CO levels, resulted in similar findings: we observed a trend of increasing beta coefficients from the 75th–95th percentiles of CO, with the beta coefficients reaching statistical significance around the 90th percentile (Fig. 4). The fitted coefficients from models using this definition of CO were slightly smaller than in the primary analysis.

### Effect of Cookstove intervention on CO

**Fig. 5:**
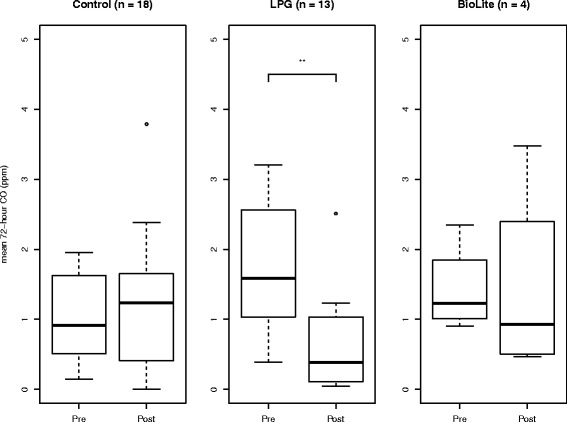
Boxplots of mean 72-h personally-monitored CO before and after a stove intervention. Dark lines indicate median values before and after the intervention. Only the group receiving the LPG stove intervention demonstrates a significant post-intervention reduction in CO

Thirty five individuals had valid CO data in both the pre-intervention and post-intervention exposure monitoring sessions: 18 in the Control group, 13 who received the LPG intervention, and 4 in the BioLite group. Pre-intervention mean 72-h CO levels were 1.04 ppm (Control), 1.74 (LPG), and 1.43 (BioLite); with post-intervention levels of 1.55 ppm (Control), 0.63 ppm (LPG), and 1.45 ppm (BioLite). Among the three groups, only the LPG group showed a significant reduction in mean CO level following the intervention (Fig. 5).

### Effect of Cookstove intervention on BP

Forty one individuals had valid BP data in both of the two BP measurement sessions and could be included in this analysis. Of these, 25 were in the ABPM group and 16 in the HBPM group, while 21 were in the intervention group and 20 were in the control group. Pre- and post-intervention SBP and DBP for each group can be found in Table 4. Although there were small differences in SBP and DBP depending on the type of BP assessment (ABPM vs. HBPM, Table 4), BP assessment method was not significantly associated with the amount of BP change (t-test *p*-value for change in SBP = 0.74; for change in DBP = 0.75).

**Table 4:** Mean [SD] SBP and DBP by intervention status and BP measurement group

			Intervention status			
			Control (*n* = 20)		Intervention (*n* = 21)	
			Round 1 (pre-intervention)	Round 2 (post-intervention)	Round 1 (pre-intervention)	Round 2 (post-intervention)
BP Measurement Group	ABP (*n* = 25)	SBP	105.3 [7.9]	105.2 [5.0]	108.3 [4.9]	107.8 [6.7]
		DBP	63.3 [5.5]	62.0 [4.8]	62.1 [4.5]	62.5 [4.9]
	Home BP (*n* = 16)	SBP	98.6 [4.0]	101.3 [8.3]	99.8 [10.4]	97.7 [7.5]
		DBP	63.0 [2.6]	65.8 [5.7]	66.9 [6.7]	64.8 [4.3]

**Fig. 6:**
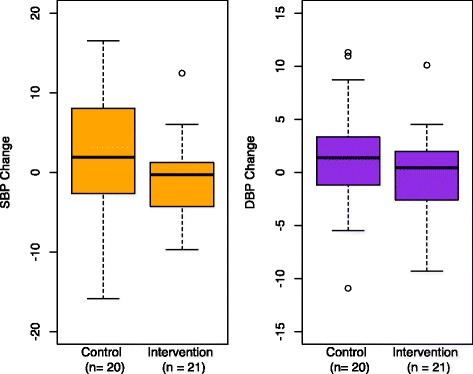
SBP/DBP change (Session 2 – Session 1) by intervention status. Negative values indicate session 2 (post-intervention) BP was lower than session 1 BP; positive values indicate the reverse

Combining data from the ABPM and HBPM groups, we analyzed the change in awake BP before and after the cookstove intervention. We observed a non-significant trend toward lower post-cookstove-intervention SBP and DBP in the intervention groups (Fig. 6 and Table 5). This result was larger for SBP than DBP in both unadjusted and adjusted models. In the adjusted analysis (Table 5; full model results available in the Additional file 1: Table S3), the coefficient for the cookstove intervention was −2.1 [−6.6, 2.4] for the change in SBP (the intervention was associated with a change in SBP that was 2.1 mmHg lower than the change in SBP among controls, holding covariates constant), while the cookstove intervention coefficient was −0.1 [−3.2, 3.0] for the change in DBP. When we restricted the “intervention” group to those women who received LPG stoves (excluding the five BioLite stove recipients), and/or analyzed the data separately by BP monitoring type, the results were similar, with the exception that there appeared to be no relationship between the intervention and SBP within the 16 women in the home BP group alone (Additional file 1: Table S4).

**Table 5:** Within-subject change in awake SBP and DBP (post-intervention minus pre-intervention) by intervention status (*n* = 41)

	Change in SBP		Change in DBP	
	Unadjusted	Adjusted^a^	Unadjusted	Adjusted^a^
Coefficient for Cookstove Intervention^a^	−3.5 [−7.7, 0.75]	−2.1 [−6.6, 2.4]	−1.5 [−4.6, 1.5]	−0.1 [−3.2, 3.0]

^a^Adjusted results are from linear regression models adjusted for gestational age and type of BP assessment (ABPM vs. HPBM)

#### Additional results

##### ABPM feasibility and validity

98.1% (53 out of 54) of the expected 24-h ABPM sessions were successfully completed (one woman traveled out of the study region prior to the second ABPM session). 92.5% of these completed ABP sessions were valid according to IDACO criteria. Within valid ABP sessions, the mean [SD] number of BP readings was 49.9 [10.9] (awake period: 30.7 [9.0]; asleep period: 19.2 [4.0]), with mean session duration of 23.1 [1.6] hours.

The HBPM protocol was also successful. In the HBPM group, 97.1% (33 out of 34) of the expected 5-day sessions were conducted (one woman left the GRAPHS trial due to early pregnancy termination). 99% of the planned 10 measurements per woman were obtained within these completed Home BP sessions, with a mean [SD] number of readings per 5-day session of 9.9 [0.3] (mornings: 4.9 [0.2]; evenings: 4.9 [0.2]).

##### Overall BP and ABPM-specific BP findings

All women in the study had mean [SD] BP levels in the normotensive range, as follows. ABPM group: SBP 106.4 [5.8]; DBP 62.2 [4.7]. HBPM group: SBP 100.0 [8.6], DBP 65.5 [5.5]. None of the women had average BP above the thresholds for hypertension as outlined in Table 1.

Because ABPM records BP values during sleeping as well as waking hours, additional parameters having to do with the diurnal cycle of BP can be calculated from ABPM records (see Table 1). None of the ABPM results indicated awake ambulatory hypertension, while two women demonstrated nocturnal hypertension during one of their two ABPM sessions only. Eighteen women demonstrated a non-dipping pattern, defined as a nocturnal decline in SBP ≤ 10%: 10 women demonstrated the non-dipping pattern during both ABPM sessions, while an additional 8 women demonstrated the non-dipping pattern during a single ABPM session only (Table 6). None of these parameters were associated either with intervention status or CO exposure.

**Table 6:** ABP parameters across 49 valid ABPM sessions

Number of sessions indicating	Count (%)
• Elevated 24 h BP (hypertension)	0
• Awake ambulatory hypertension	0
• Nocturnal hypertension	2 (4%)
• Non-dipping pattern	28 (57%)
Average values	Mean [SD]
• Mean 24-h SBP	106.4 [5.8]
• Mean 24-h DBP	62.2 [4.7]
• Awake SBP	110.5 [5.9]
• Awake DBP	66.7 [5.0]
• Asleep SBP	100.4 [7.9]
• Asleep DBP	55.8 [6.4]

## Discussion

Using 24-h ABPM and concurrent personal monitoring of carbon monoxide, we showed that exposure to CO levels above 4.1 ppm in the 2 h prior to blood pressure measurement was significantly associated with transient increases in both SBP and DBP in a small cohort of pregnant women in Ghana. The observed effect was a mean increase of 4.3 mmHg in hourly SBP and 4.5 mmHg in hourly DBP. In sensitivity analyses using alternate definitions of CO, we observed that peak CO exposure above the 75th percentile of the exposure distribution was consistently associated with increased SBP and DBP.

Further, in an analysis of the change in BP before and after a cookstove intervention as compared to control, we observed that the cookstove intervention was associated with a reduction in SBP and DBP among pregnant women who received the improved cookstoves, while SBP and DBP increased slightly in pregnant control women over the same time period. These results were in the hypothesized direction but were not statistically significant. Restricting the analysis to women who received the LPG stove intervention led to slightly larger estimates of effect.

While BP is inherently variable, the fact that HAP exposure may be associated with transient increases in BP among normotensive individuals relates directly to theories about the development of essential hypertension. Current research on the pathogenesis of chronic hypertension suggests that modifications in vasculature resulting from intermittent increased local load pressure in the arteries and arterioles lead over time to increased arterial stiffness and peripheral vascular resistance to blood flow [43–45]. If an environmental trigger repetitively induces transient elevations in blood pressure, this may set the stage for the eventual development of chronic hypertension in a susceptible individual. A transient effect of HAP exposure on BP, as seen here, thus suggests a plausible mechanistic explanation for the associations that have been observed between HAP exposure and elevations in BP [7, 17] and between cookstove interventions and reductions in BP [20]. Eventually, this BP effect could be a contributing factor to CVD and help explain associations between chronic HAP exposure and adverse cardiovascular diagnoses [3, 4]. If further studies confirm this mechanistic pathway between HAP and hypertension/CVD, it is importantly amenable to intervention. We observed a trend in this study toward lower post-intervention BP among women receiving improved cookstoves.

These results additionally indicate that 24-h ABPM is a well-tolerated and feasible method of BP assessment in pregnant women in this predominately rural West African setting. We achieved a 92.5% validity rate for the 24-h ABPM sessions completed here; and these results compare favorably to results in other populations. For example, an analysis from the IDACO study, a multinational project using ABPM among 12,711 people drawn from 12 population cohorts from Europe, Asia, and South America, reported a validity rate of 84.5% using the same criteria applied here [34].

Twenty four hour ABPM also led to some unexpected findings about diurnal BP patterns in this cohort of Ghanaian women. BP normally dips 10–20% during sleep, driven largely by inactivity [46]. Notably, 57% of the ABPM sessions in this study population were characterized by a non-dipping nocturnal pattern, defined as a nocturnal decline in SBP ≤ 10% as compared to waking values. The prevalence of non-dipping observed in this cohort is greater than has been reported in several prominent observational studies of ABPM parameters, including 27.8% non-dipping rate reported in the PIUMA study among 959 individuals with ambulatory hypertension in Italy [47], and the 29.4% non-dipping rate reported within the IDACO cohort of 7458 individuals [48]. Non-dipping has been associated with adverse cardiovascular outcomes in a variety of European, American, and Asian cohorts [46, 49], including associations among pregnant women with pregnancy-induced hypertension [50] and increased risk of endothelial damage in preeclampsia [51]. Studies in the United States point to a potential racial and ethnic component of non-dipping patterns, with a larger prevalence of non-dipping SBP observed among African-Americans as compared to other racial groups [52, 53]. The high non-dipping rate reported in this West African cohort may indicate that this question deserves further study in relation to hypertension and CVD in African populations.

### Limitations

The small size of our study limited our ability to draw generalizable conclusions. While the sample size of 44 women was adequately powered for evaluating the hourly effect of CO on BP, due to missing data we ended up having a smaller sample of data to analyze for the acute CO-BP relationship (20 24-h ABPM/CO monitoring sessions). We were also underpowered to test the overall effect of the cookstove intervention. Larger studies will be needed to corroborate the results observed here: both of a threshold effect of acute CO exposure on BP increases and the trend toward lower post-intervention BP among women receiving an improved cookstove. As this study focused on pregnant women, the results may be most pertinent to other cohorts of pregnant women, who are a particularly vulnerable subgroup for hypertensive complications. The implications for cohorts of men and non-pregnant women are unclear.

Another limitation is that the exposure data available was for CO, and not PM, for which a stronger body of literature exists for a relationship with blood pressure and other cardiovascular outcomes. CO and PM correlate well in some settings using biomass fuels [20, 21], but not in others [23, 54]. While CO and PM both are major constituents of household air pollution from biomass burning, they cannot simply be considered proxies for each other. We therefore cannot be certain whether it is CO, PM, or another pollutant that co-varies with CO that caused the differences observed.

Finally, we did not have data on some important potential covariates that might either confound or modify the relationship between CO and BP, such as diet, ambient temperature, and physical activity. In particular, cooking in this region can be quite physically demanding, requiring vigorous stirring of thick porridges and pounding of starchy root vegetables. Since exercise influences blood pressure [55], is therefore possible that the association seen between CO exposure and subsequent increases in BP was due in part to increased physical activity during cooking. It should be noted, however, that other common foods from this region are boiled or stewed and thus require less exertion to prepare. Furthermore, many other activities of daily life in this region besides cooking are physically demanding, such as washing clothes, farming, and gathering firewood – all of which are activities the women may have engaged in during their 24-h ABPM sessions. Nonetheless, future studies in settings such as this one should incorporate more detailed physical activity monitoring and/or time-activity tracking to better disentangle the relationships between HAP exposure, physical activity, and changes in BP.

## Conclusions

Using concurrent ABPM and personal CO monitoring, we found evidence of an acute relationship between exposure to CO and increased BP at the high end of the CO exposure distribution. CO above 4.1 ppm up to 2 h prior to BP measurement was significantly associated with increases in SBP and DBP that were over 4 mmHg respectively.

This study demonstrated that 24-h ABPM is a well-tolerated and feasible method of BP assessment among pregnant women in a rural African setting. ABPM provides a wealth of information on diurnal patterns of BP that can be related to cardiovascular health status, and is a promising tool to investigate the cardiovascular effects of cookstove interventions in low- and middle-income countries.

## Additional file

Additional file 1: Table S1. Results from regression models estimating the effect of different lags of CO on SBP and DBP. **Figure S1.** Beta coefficients for effect of hourly CO exposure on hourly BP. **Table S2.** Association of hourly peak CO exposure with hourly SBP and DBP, full adjusted linear regression results. *N* = 20 24-h monitoring sessions among 19 women. **Table S3.** Change in awake SBP and DBP by intervention status, full adjusted linear regression results (*n* = 41 women). **Table S4.** Results of sensitivity analyses investigating the relationship between intervention status and change in BP among subgroups: those in the Home BP arm vs. the ABP arm, and defining the “intervention” as any intervention cookstove (BioLite or LPG) or solely among the LPG recipients. (DOCX 138 kb)

## Abbreviations

ABP: Ambulatory blood pressure
ABPM: Ambulatory blood pressure monitoring
BP: Blood pressure
CO: Carbon monoxide
CVD: Cardiovascular disease
DBP: Diastolic blood pressure
ESH: European Society of Hypertension
GRAPHS: Ghana randomized air pollution and health study
HAP: Household Air Pollution
HBPM: Home Blood Pressure Monitoring
IDACO: International database of ambulatory blood pressure in relation to cardiovascular outcome
LPG: Liquefied petroleum gas
PM: Particulate matter
SBP: Systolic blood pressure

## References

[1] Lim SS, Vos T, Flaxman AD, Danaei G, Shibuya K, Adair-Rohani H (2012). A comparative risk assessment of burden of disease and injury attributable to 67 risk factors and risk factor clusters in 21 regions, 1990–2010: a systematic analysis for the Global burden of disease study 2010. Lancet.

[2] Institute for Health Metrics and Evaluation. Global Burden of Disease Country Profiles | Ghana. 2013. http://www.healthdata.org/sites/default/files/files/country_profiles/GBD/ihme_gbd_country_report_ghana.pdf.

[3] Lee M-S, Hang J, Zhang F, Dai H, Su L, Christiani DC (2012). In-home solid fuel use and cardiovascular disease: a cross-sectional analysis of the shanghai Putuo study. Environ Health.

[4] Fatmi Z, Coggon D, Kazi A, Naeem I, Kadir MM, Sathiakumar N (2014). Solid fuel use is a major risk factor for acute coronary syndromes among rural women: a matched case control study. Public Health.

[5] Mitter SS, Vedanthan R, Islami F, Pourshams A, Khademi H, Kamangar F (2016). Household fuel use and cardiovascular disease mortality: Golestan cohort study. Circulation.

[6] Brook RD, Rajagopalan S (2009). Particulate matter, air pollution, and blood pressure. J Am Soc Hypertens JASH.

[7] McCracken JP, Wellenius GA, Bloomfield GS, Brook RD, Tolunay HE, Dockery DW (2012). Household air pollution from solid fuel use: evidence for links to CVD. Glob Heart.

[8] McCracken JP, Smith KR, Díaz A, Mittleman MA, Schwartz J (2007). Chimney stove intervention to reduce long-term wood smoke exposure lowers blood pressure among Guatemalan women. Environ Health Perspect.

[9] Dutta A, Mukherjee B, Das D, Banerjee A, Ray MR (2011). Hypertension with elevated levels of oxidized low-density lipoprotein and anticardiolipin antibody in the circulation of premenopausal Indian women chronically exposed to biomass smoke during cooking. Indoor Air.

[10] Norris C, Goldberg MS, Marshall JD, Valois M-F, Pradeep T, Narayanswamy M (2016). A panel study of the acute effects of personal exposure to household air pollution on ambulatory blood pressure in rural Indian women. Environ Res.

[11] Wylie BJ, Singh MP, Coull BA, Quinn A, Yeboah-Antwi K, Sabin L, et al. Association between wood cooking fuel and maternal hypertension at delivery in central East India. Hypertens Pregnancy. 2015;34(3):355–68. 10.3109/10641955.2015.1046604 PMC4674790 26153626

[12] Clark ML, Bazemore H, Reynolds SJ, Heiderscheidt JM, Conway S, Bachand AM (2011). A baseline evaluation of traditional cook stove smoke exposures and indicators of cardiovascular and respiratory health among Nicaraguan women. Int J Occup Environ Health.

[13] Baumgartner J, Zhang Y, Schauer JJ, Huang W, Wang Y, Ezzati M (2014). Highway proximity and black carbon from cookstoves as a risk factor for higher blood pressure in rural China. Proc Natl Acad Sci.

[14] Burroughs Pena M, Romero KM, Velazquez EJ, Davila-Roman VG, Gilman RH, Wise RA, et al. Relationship Between Daily Exposure to Biomass Fuel Smoke and Blood Pressure in High-Altitude Peru. Hypertension. 2015;65. doi:10.1161/HYPERTENSIONAHA.114.04840. 10.1161/HYPERTENSIONAHA.114.04840 PMC4466100 25753976

[15] Painschab MS, Davila-Roman VG, Gilman RH, Vasquez-Villar AD, Pollard SL, Wise RA (2013). Chronic exposure to biomass fuel is associated with increased carotid artery intima-media thickness and a higher prevalence of atherosclerotic plaque. Heart.

[16] Baumgartner J, Schauer JJ, Ezzati M, Lu L, Cheng C, Patz JA (2011). Indoor air pollution and blood pressure in adult women living in rural China. Environ Health Perspect.

[17] Quinn AK, Ae-Ngibise KA, Jack DW, Boamah EA, Enuameh Y, Mujtaba MN (2016). Association of Carbon Monoxide exposure with blood pressure among pregnant women in rural Ghana: evidence from GRAPHS. Int J Hyg Environ Health.

[18] Naeher LP, Brauer M, Lipsett M, Zelikoff JT, Simpson CD, Koenig JQ (2007). Woodsmoke health effects: a Review. Inhal Toxicol.

[19] Dionisio KL, Howie S, Fornace KM, Chimah O, Adegbola RA, Ezzati M (2008). Measuring the exposure of infants and children to indoor air pollution from biomass fuels in the Gambia. Indoor Air.

[20] McCracken JP, Schwartz J, Diaz A, Bruce N, Smith KR (2013). Longitudinal relationship between personal CO and personal PM2.5 among women cooking with Woodfired Cookstoves in Guatemala. PLoS One.

[21] Pollard SL, Williams DL, Breysse PN, Baron PA, Grajeda LM, Gilman RH (2014). A cross-sectional study of determinants of indoor environmental exposures in households with and without chronic exposure to biomass fuel smoke. Environ Health Glob Access Sci Source.

[22] Clark ML, Peel JL, Balakrishnan K, Breysse PN, Chillrud SN, Naeher LP (2013). Health and household air pollution from solid fuel use: the need for improved exposure assessment. Environ Health Perspect.

[23] Klasen EM, Wills B, Naithani N, Gilman RH, Tielsch JM, Chiang M (2015). Low correlation between household carbon monoxide and particulate matter concentrations from biomass-related pollution in three resource-poor settings. Environ Res.

[24] de Paula SU, Braga ALF, Giorgi DMA, Pereira LAA, Grupi CJ, Lin CA (2005). Effects of air pollution on blood pressure and heart rate variability: a panel study of vehicular traffic controllers in the city of São Paulo. Brazil Eur Heart J.

[25] Jack DW, Asante KP, Wylie BJ, Chillrud SN, Whyatt RM, Ae-Ngibise KA (2015). Ghana randomized air pollution and health study (GRAPHS): study protocol for a randomized controlled trial. Trials.

[26] Mancia G, Fagard R, Narkiewicz K, Redón J, Zanchetti A, Böhm M (2013). 2013 ESH/ESC guidelines for the management of arterial hypertension: the task force for the management of arterial hypertension of the European Society of Hypertension (ESH) and of the European Society of Cardiology (ESC). J Hypertens.

[27] Shimbo D, Abdalla M, Falzon L, Townsend RR, Muntner P (2015). Role of ambulatory and home blood pressure monitoring in clinical practice: a narrative Review. Ann Intern Med.

[28] Pickering TG, Hall JE, Appel LJ, Falkner BE, Graves JW, Hill MN (2005). Recommendations for blood pressure measurement in humans: an AHA scientific statement from the council on high blood pressure research professional and Public education subcommittee. J Clin Hypertens Greenwich Conn.

[29] Chalmers J, Arima H, Harrap S, Touyz RM, Park JB (2013). Global survey of current practice in management of hypertension as reported by societies affiliated with the International Society of hypertension. J Hypertens.

[30] Boamah EA, Asante K, Ae-Ngibise K, Kinney PL, Jack DW, Manu G (2014). Gestational age assessment in the Ghana randomized air pollution and health study (GRAPHS): ultrasound capacity building, Fetal biometry protocol development, and ongoing Quality control. JMIR Res Protoc.

[31] Association for the Advancement of Medical Instrumentation (1993). American national standard. Electronic or automated sphygmomanometers. ANSI/AAMI SP 10–1992.

[32] Shennan AH, Kissane J, de Swiet M (1993). Validation of the SpaceLabs 90207 ambulatory blood pressure monitor for use in pregnancy. Br J Obstet Gynaecol.

[33] Topouchian J, Agnoletti D, Blacher J, Youssef A, Chahine MN, Ibanez I (2014). Validation of four devices: Omron M6 comfort, Omron HEM-7420, Withings BP-800, and Polygreen KP-7670 for home blood pressure measurement according to the European Society of Hypertension International Protocol. Vasc Health Risk Manag.

[34] Li Y, Wei F-F, Thijs L, Boggia J, Asayama K, Hansen TW (2014). Ambulatory hypertension subtypes and 24-hour systolic and diastolic blood pressure as distinct outcome predictors in 8341 untreated people recruited from 12 populations. Circulation.

[35] O’Brien E, Parati G, Stergiou G, Asmar R, Beilin L, Bilo G (2013). European Society of Hypertension position paper on ambulatory blood pressure monitoring. J Hypertens.

[36] Ezzati M, Saleh H, Kammen DM (2000). The contributions of emissions and spatial microenvironments to exposure to indoor air pollution from biomass combustion in Kenya. Environ Health Perspect.

[37] Ezzati M, Kammen DM (2001). Indoor air pollution from biomass combustion and acute respiratory infections in Kenya: an exposure-response study. Lancet.

[38] Urch B, Silverman F, Corey P, Brook JR, Lukic KZ, Rajagopalan S (2005). Acute blood pressure responses in healthy adults during controlled air pollution exposures. Environ Health Perspect.

[39] Langrish JP, Mills NL, Chan JK, Leseman DL, Aitken RJ, Fokkens PH (2009). Beneficial cardiovascular effects of reducing exposure to particulate air pollution with a simple facemask. Part Fibre Toxicol.

[40] Cosselman KE, Krishnan RM, Oron AP, Jansen K, Peretz A, Sullivan JH (2012). Blood pressure response to controlled diesel exhaust exposure in human subjects. Hypertension.

[41] Bellavia A, Urch B, Speck M, Brook RD, Scott JA, Albetti B (2013). DNA hypomethylation, ambient particulate matter, and increased blood pressure: findings from controlled human exposure experiments. J Am Heart Assoc.

[42] Creasy RK, Resnik R, Iams JD, Lockwood CJ, Moore T, Greene MF. Creasy and Resnik’s Maternal-Fetal Medicine: Principles and Practice, 7th Edition (Elsevier Saunders, 2014).

[43] Intengan HD, Schiffrin EL (2000). Structure and mechanical properties of resistance arteries in hypertension: role of adhesion molecules and extracellular matrix determinants. Hypertension.

[44] Eiken O, Kölegård R (2011). Repeated exposures to moderately increased intravascular pressure increases stiffness in human arteries and arterioles. J Hypertens.

[45] Eiken O, Mekjavic IB, Kölegård R (2014). Blood pressure regulation V: in vivo mechanical properties of precapillary vessels as affected by long-term pressure loading and unloading. Eur J Appl Physiol.

[46] Hansen TW, Li Y, Boggia J, Thijs L, Richart T, Staessen JA (2011). Predictive role of the nighttime blood pressure. Hypertension.

[47] Verdecchia P, Porcellati C, Schillaci G, Borgioni C, Ciucci A, Battistelli M (1994). Ambulatory blood pressure. An independent predictor of prognosis in essential hypertension. Hypertension.

[48] Boggia J, Li Y, Thijs L, Hansen TW, Kikuya M, Björklund-Bodegård K (2007). Prognostic accuracy of day versus night ambulatory blood pressure: a cohort study. Lancet Lond Engl.

[49] Parati G, Valentini M (2006). Prognostic relevance of blood pressure variability. Hypertension.

[50] Kärkkäinen H, Saarelainen H, Laitinen T, Heiskanen N, Valtonen P, Laitinen T (2014). Ambulatory arterial stiffness index and nocturnal blood pressure dipping in pregnancies complicated by hypertension. Clin Physiol Funct Imaging.

[51] Bouchlariotou S, Liakopoulos V, Dovas S, Giannopoulou M, Kiropoulos T, Zarogiannis S (2008). Nocturnal hypertension is associated with an exacerbation of the endothelial damage in preeclampsia. Am J Nephrol.

[52] Jehn ML, Brotman DJ, Appel LJ (2008). Racial differences in diurnal blood pressure and heart rate patterns: results from the dietary approaches to stop hypertension (DASH) trial. Arch Intern Med.

[53] Sherwood A, Routledge FS, Wohlgemuth WK, Hinderliter AL, Kuhn CM, Blumenthal JA (2011). BLOOD PRESSURE DIPPING: ETHNICITY, SLEEP QUALITY AND SYMPATHETIC NERVOUS SYSTEM ACTIVITY. Am J Hypertens.

[54] Dionisio KL, Howie SRC, Dominici F, Fornace KM, Spengler JD, Adegbola RA (2012). Household concentrations and exposure of children to particulate matter from biomass fuels in the Gambia. Environ Sci Technol.

[55] Joyner MJ, Limberg JK (2014). Blood pressure regulation: every adaptation is an integration?. Eur J Appl Physiol.

