# Supplementary material for: Ambulatory monitoring demonstrates an acute association between cookstove-related carbon monoxide and blood pressure in a Ghanaian cohort
Source: Environ Health. 2017 Jul 21;16:76. doi: 10.1186/s12940-017-0282-9 (PMC5521137; doi:10.1186/s12940-017-0282-9)
Supplement: Additional file 1: Table S1. — Results from regression models estimating the effect of different lags of CO on SBP and DBP. Figure S1. Beta coefficients for effect of hourly CO exposure on hourly BP. Table S2. Association of hourly peak CO exposure with hourly SBP and DBP, full adjusted linear regression results. N = 20 24-h monitoring sessions among 19 women. Table S3. Change in awake SBP and DBP by intervention status, full adjusted linear regression results (n = 41 women). Table S4. Results of sensitivity analyses investigating the relationship between intervention status and change in BP among subgroups: those in the Home BP arm vs. the ABP arm, and defining the “intervention” as any intervention cookstove (BioLite or LPG) or solely among the LPG recipients. (DOCX 138 kb) [file 12940_2017_282_MOESM1_ESM.docx]

## Additional file

**Figure S1**. Beta coefficients for effect of hourly CO exposure on hourly BP. Data points and error bars represent the estimated changes and 95% confidence intervals in SBP and DBP associated with a 1-IQR (1.64ppm) increase in 1- to 8-hour single hour lags of personally-monitored CO exposure. n = 20 24-hour ABPM sessions. All models are adjusted for the awake period, peri-waking morning period, and ABPM session.

**Table S1.** Results from regression models estimating the effect of different lags of CO on SBP and DBP (90^th^ percentile CO at lag 0, 1, and 2 hours behind BP).

| **SBP (mmHg)** | | | |  | **DBP (mmHg)** | | | |
| --- | --- | --- | --- | --- | --- | --- | --- | --- |
| **Model SBP1** |  |  |  |  | **Model DBP1** |  |  |  |
|  | Coefficient | SE | p-value |  |  | Coefficient | SE | p-value |
| Intercept | 101.64 | 1.56 | < 0.0001 |  | Intercept | 56 | 1.23 | < 0.0001 |
| **CO_90_lag0** | **1.66** | **1.49** | **0.26** |  | **CO_90_lag0** | **1.75** | **1.27** | **0.17** |
| Waking hours | 9.69 | 1.17 | < 0.0001 |  | Waking hours | 10.5 | 1.04 | < 0.0001 |
| Morning | 6.29 | 1.56 | 0.0001 |  | Morning | 7.41 | 1.36 | < 0.0001 |
|  |  |  |  |  |  |  |  |  |
| **Model SBP2** | | | |  | **Model DBP2** | | | |
|  | Coefficient | SE | p-value |  |  | Coefficient | SE | p-value |
| Intercept | 101.36 | 1.59 | < 0.0001 |  | Intercept | 55.77 | 1.23 | < 0.0001 |
| **CO_90_lag1** | **3.3** | **1.49** | **0.03** |  | **CO_90_lag1** | **3.11** | **1.27** | **0.01** |
| Waking hours | 9.53 | 1.17 | < 0.0001 |  | Waking hours | 10.37 | 1.02 | < 0.0001 |
| Morning | 6.4 | 1.57 | 0.0001 |  | Morning | 7.58 | 1.35 | < 0.0001 |
|  |  |  |  |  |  |  |  |  |
| **Model SBP3** | | | |  | **Model DBP3** | | | |
|  | Coefficient | SE | p-value |  |  | Coefficient | SE | p-value |
| Intercept | 101.53 | 1.57 | < 0.0001 |  | Intercept | 55.96 | 1.27 | < 0.0001 |
| **CO_90_lag2** | -0.63 | 1.54 | 0.68 |  | **CO_90_lag2** | -0.93 | 1.32 | 0.48 |
| Waking hours | 9.58 | 1.18 | < 0.0001 |  | Waking hours | 10.53 | 1.05 | < 0.0001 |
| Morning | 6.26 | 1.58 | 0.0001 |  | Morning | 7.54 | 1.37 | < 0.0001 |
|  |  |  |  |  |  |  |  |  |
| **Model SBP4** | | | |  | **Model DBP4** | | | |
|  | Coefficient | SE | p-value |  |  | Coefficient | SE | p-value |
| Intercept | 101.4 | 1.58 | < 0.0001 |  | Intercept | 55.81 | 1.25 | < 0.0001 |
| **CO_90_lag0** | **0.37** | **1.76** | **0.83** |  | **CO_90_lag0** | **0.37** | **1.5** | **0.8** |
| **CO_90_lag1** | **3.47** | **1.67** | **0.04** |  | **CO_90_lag1** | **3.53** | **1.42** | **0.01** |
| **CO_90_lag2** | **-1.07** | **1.57** | **0.5** |  | **CO_90_lag2** | **-1.38** | **1.34** | **0.3** |
| Waking hours | 9.23 | 1.21 | < 0.0001 |  | Waking hours | 10.2 | 1.07 | < 0.0001 |
| Morning | 6.29 | 1.58 | 0.0001 |  | Morning | 7.52 | 1.37 | < 0.0001 |

Models SBP1, SBP2, and SBP3; and DBP1, DBP2, and DBP3: models incorporating singly lagged CO variables. The effect of 90^th^ percentile CO is positive on BP at lags 0 and 1, with the effect stronger at lag 2. The effect goes away at lag 2 hours. Models SBP4 and DBP4: models with multiple lags of CO in the same model. Results are consistent with the single-variable models.

**Table S2.** Association of hourly peak CO exposure with hourly SBP and DBP, full adjusted linear regression results. N = 20 24-hour monitoring sessions among 19 women.

| **Outcome** |  | **Coefficient*** | **p-value** |
| --- | --- | --- | --- |
| **SBP (mmHg)** | **Intercept** | 101.46 | <0.0001 |
|  | **Peak CO (2-hour moving average)** | 4.25 | 0.008 |
|  | **Waking hours** | 9.19 | <0.0001 |
|  | **Morning** | 6.3 | 0.0001 |
|  |  |  |  |
| **DBP (mmHg)** | **Intercept** | 55.83 | <0.0001 |
|  | **Peak CO (2-hour moving average)** | 4.54 | 0.0004 |
|  | **Waking hours** | 10.02 | <0.0001 |
|  | **Morning** | 7.41 | <0.0001 |

* The coefficient is the estimated fixed-effect difference in BP associated with peak hourly CO exposure (defined as a binary indicator of CO meeting or exceeding the 90^th^ percentile of a two-hour moving average of CO, or 4.1 ppm) versus all other CO exposure, adjusted for self-reported awake hours and for the 2-hour peri-waking “morning” period. Model is a multilevel linear regression model with a random intercept for each 24-hour monitoring session. Estimates incorporate an autoregressive (AR1) correlation structure.

**Table S3.** Change in awake SBP and DBP by intervention status, full adjusted linear regression results (n=41 women).

|  |  | **Coefficient** | **p-value** |
| --- | --- | --- | --- |
| **Change in SBP (mmHg)** | **Intercept** | -5.1 | 0.32 |
|  | **Intervention** | -2.1 | 0.35 |
|  | **Gestational age** | 0.4 | 0.11 |
|  | **Type of BP Measurement (HBPM)** | -0.7 | 0.74 |
|  |  |  |  |
| **Change in DBP (mmHg)** | **Intercept** | -6.8 | 0.07 |
|  | **Intervention** | -0.1 | 0.95 |
|  | **Gestational age** | 0.5 | 0.02 |
|  | **Type of BP Measurement (HBPM)** | -0.5 | 0.75 |

**Table S4.** Results of sensitivity analyses investigating the relationship between intervention status and change in BP among subgroups: those in the Home BP arm vs. the ABP arm, and defining the “intervention” as any intervention cookstove (BioLite or LPG) or solely among the LPG recipients (too few women received the BioLite stove to conduct subgroup analyses in this group).

|  | **Data** | **n** | **Coefficient for intervention** | **95% CI** | **p-value** |
| --- | --- | --- | --- | --- | --- |
| **SBP** | Home BP and ABP; intervention = any improved stove | 41 | -2.1 | [-6.6, 2.4] | 0.35 |
|  | Home BP and ABP; intervention = LPG only | 36 | -2.5 | [-7.58, 2.58] | 0.33 |
|  | ABP only; intervention = any improved stove | 25 | -2.5 | [-8.4, 3.4] | 0.39 |
|  | ABP only; intervention = LPG only | 20 | -2.7 | [-9.7, 4.4] | 0.43 |
|  | Home BP only; intervention = LPG only* | 16 | 0.2 | [-11.3, 11.9] | 0.97 |
| **DBP** | Home BP and ABP; intervention = any improved stove | 41 | -0.1 | [-3.2, 3.0] | 0.95 |
|  | Home BP and ABP; intervention = LPG only | 36 | -0.8 | [-4.43, 2.83] | 0.65 |
|  | ABP only; intervention = any improved stove | 25 | 0.7 | [-3.2, 4.6] | 0.72 |
|  | ABP only; intervention = LPG only | 20 | 0.2 | [-4.4, 4.9] | 0.91 |
|  | Home BP only; intervention = LPG only* | 16 | -0.6 | [-9.4, 8.2] | 0.88 |

* All women in the home BP group received the LPG intervention; none received BioLite stoves.
